# Cardiovascular and metabolic comorbidities in patients with thyroid nodules: the impact of incidental diagnosis

**DOI:** 10.1007/s40618-023-02191-4

**Published:** 2023-09-13

**Authors:** L. Croce, R. M. Ruggeri, C. Cappelli, C. Virili, F. Coperchini, M. Laganà, P. Costa, M. Dal Molin, S. Chytiris, F. Magri, L. Chiovato, M. Centanni, S. Cannavò, M. Rotondi

**Affiliations:** 1 Department of Internal Medicine and Therapeutics, University of Pavia, Via S. Maugeri 4, 27100 Pavia (PV), Italy; 2 Unit of Endocrinology and Metabolism, Laboratory for Endocrine Disruptors, Istituti Clinici Scientifici Maugeri IRCCS, 27100 Pavia (PV), Italy; 3 Department of Human Pathology and Childhood “G. Barresi” (DETEV), University of Messina, 98125 Messina (ME), Italy; 4 Department of Clinical and Experimental Sciences, SSD Medicina ad indirizzo Endocrino-Metabolico, University of Brescia, ASST Spedali Civili di Brescia, 25123 Brescia (BS), Italy; 5 Endocrinology Section, Department of Medico-Surgical Sciences and Biotechnologies, “Sapienza” University of Rome, 04100 Latina (LT), Italy

**Keywords:** Thyroid nodules, Cardiovascular disease, Metabolic syndrome, Incidental diagnosis

## Abstract

**Purpose:**

The prevalence of thyroid nodules (TN) in the general population has increased as screening procedures are implemented and an association with metabolic and cardiovascular disorders has been reported. The aim of this study was to investigate the reason leading to the diagnosis of TN and to compare the clinical characteristics of patients diagnosed incidentally with those of patients diagnosed for thyroid-related reasons.

**Methods:**

We designed a retrospective cross-sectional study including consecutive patients with TN from two high-volume hospital-based centers for thyroid diseases (Pavia and Messina) in Italy. Data regarding reason leading to TN diagnosis, age, sex, BMI, presence of cardio-metabolic comorbidities were collected.

**Results:**

Among the 623 enrolled subjects, the US diagnosis of TN was prompted by thyroid-related reasons in 421 (67.6%, TD group) and incidental in 202 (32.4%, ID group) with a similar distribution in the two centers (*p* = 0.960). The ID group patients were more frequently males (38.6% vs 22.1%, *p* < 0.001) and significantly older (58.9 ± 13.7 vs 50.6 ± 15.5 years, *p* < 0.001) than the TD group ones, and had a higher rate of cardiovascular comorbidities (73.8% vs 47.5%, *p* < 0.001), despite having a similar BMI (27.9 ± 5.2 vs 27.8 ± 13.5, *p* = 0.893).

**Conclusions:**

Stratification of patients with TN according to the diagnostic procedure leading to diagnosis allows a better epidemiological characterization of this inhomogeneous and large population.

## Introduction

Thyroid nodules (TN) are the most frequent condition affecting the thyroid gland, with an expected prevalence in the general population ranging from 2 to 6% (when detected by neck palpation) up to 19–35% (when detected by ultrasound) [1]. Among the described risk factors for the development of TN, iodine deficiency is undoubtedly the most universally recognized [2]; however, several other factors including increasing age, radiation exposure during childhood, and familiarity for TN also play a role [3]. More recently, it was reported that also insulin resistance could increase the risk of developing a thyroid nodule [4–8].

The epidemiology of nodular thyroid diseases dramatically changed in the last 20 years, due to the widespread use of thyroid ultrasound. Before the introduction of ultrasound, TN were diagnosed because of a palpable lump in the neck or the presence of symptoms due to airway or esophagus obstruction and/or to thyroid dysfunction. The use of ultrasound allowed the detection of even small, non-palpable TN, leading to an “epidemic” of this pathology across many developed countries. Screening procedures for unrelated conditions are also responsible for the increased detection of TN. Given the paucisymptomatic nature of TN, it is clear that the incidence of TN dramatically increases when screening procedures are implemented [9, 10]. Patients with obesity, dyslipidemia, type 2 diabetes, cardiovascular disease, and hypertension frequently undergo carotid artery ultrasound as a screening for arterial atherosclerosis.

The aim of our multicenter retrospective study was to investigate the reason leading to the diagnosis of TN and to compare the clinical characteristics of patients receiving an incidental as opposed to a clinical diagnosis.

## Materials and methods

The outpatients’ Data Bases of the Unit of Endocrinology of ICS Maugeri (Pavia, Italy) and of the Unit of Endocrinology of Messina were retrospectively searched for consecutive patients who received a diagnosis of nodular thyroid disease between December 2021 and October 2022. The two geographical areas are quite similar concerning iodine nutrition [11, 12].

The inclusion criteria were: (i) a thyroid ultrasound (US) performed in an endocrinological setting confirming the presence of TN; and (ii) the availability of information regarding the reason leading to the performance of a thyroid US.

Patients were then stratified according to the reason leading to the performance of thyroid ultrasound in two broad categories: (1) those who were addressed to thyroid ultrasound for clinical evidence or suspicion of thyroid pathology (Thyroid Disease group, TD); and (2) patients in whom TN were incidentally discovered by primarily unrelated imaging (Incidental Diagnosis Group, ID).

For the purpose of this study, the TD group was identified by evidence for one or more of the following conditions: (i) any type of thyroid function abnormality (subclinical or overt hypo-hyperthyroidism); (ii) positive tests for thyroid auto-antibodies even in the presence of normal thyroid function parameters; (iii) clinically detected TN; (iv) complaints of constrictive symptoms; and (v) screening procedures for family history of thyroid disease or any thyroid-related symptom.

The incidental group was identified by the discovery of TN by: (i) a Doppler ultrasound of carotid arteries; (ii) a computerized tomography (CT) or a Magnetic Resonance Imaging (MRI) which included the neck area; iii) a PET scan, usually performed for oncological purposes; and (iv) a neck ultrasound performed for non-thyroid conditions, including ear, nose and throat (ENT) pathologies, neck lymph-nodes enlargement, and primary hyperparathyroidism.

In any case, the diagnosis of TN was confirmed by a thyroid US performed by skilled endocrinologists. At each center, thyroid US scans were performed by the same experienced operator using a real-time US device equipped with a linear transducer operating at 7.5 MHz.

At the time of diagnosis, the following data were recorded: patient’s age (years), sex (male vs female), body mass index (kg/m^2^), and a full medical history specifically focusing on the presence of type 2 diabetes mellitus, arterial hypertension, dyslipidemia, ischemic cardiopathy, cerebral vasculopathy, and peripheral artery disease. Ultrasonographic data, including thyroid volume, number of nodules and the longest diameter of the biggest nodule were also collected.

The required sample size was calculated considering: (i) a Type I error of 0.01 and a Type II error of 0.20; (ii) an expected prevalence of incidental diagnosis of TN of 20% among our series of patients (based on preliminary data); and (iii) an expect prevalence of cardiovascular/metabolic comorbidities of around 70% in the incidental diagnosis group compared with a 50% of the non-incidental diagnosis group. The required sample size was of at least 507 patients.

All patients have signed an informed consent concerning the future use of their clinical pathological data for research purposes. This study was approved by the Ethical Committee of ICS Maugeri, Pavia (Protocol N. 2742 CE).

### Statistical analysis

Statistical analysis was performed using the SPSS Software (SPSS, Inc.). Between-groups comparisons were performed using the Student’s *t*-test for unpaired data and the Mann–Whitney *U*-test according to a normal or a non-parametric distribution. Within-group comparisons were performed using the Student’s *t*-test for paired data and the Wilcoxon’s test according to a normal or a non-parametric distribution. Frequencies among groups were performed using the *χ*^2^-test with Fisher’s correction when appropriate.

## Results

### Study participants

**Fig. 1:**
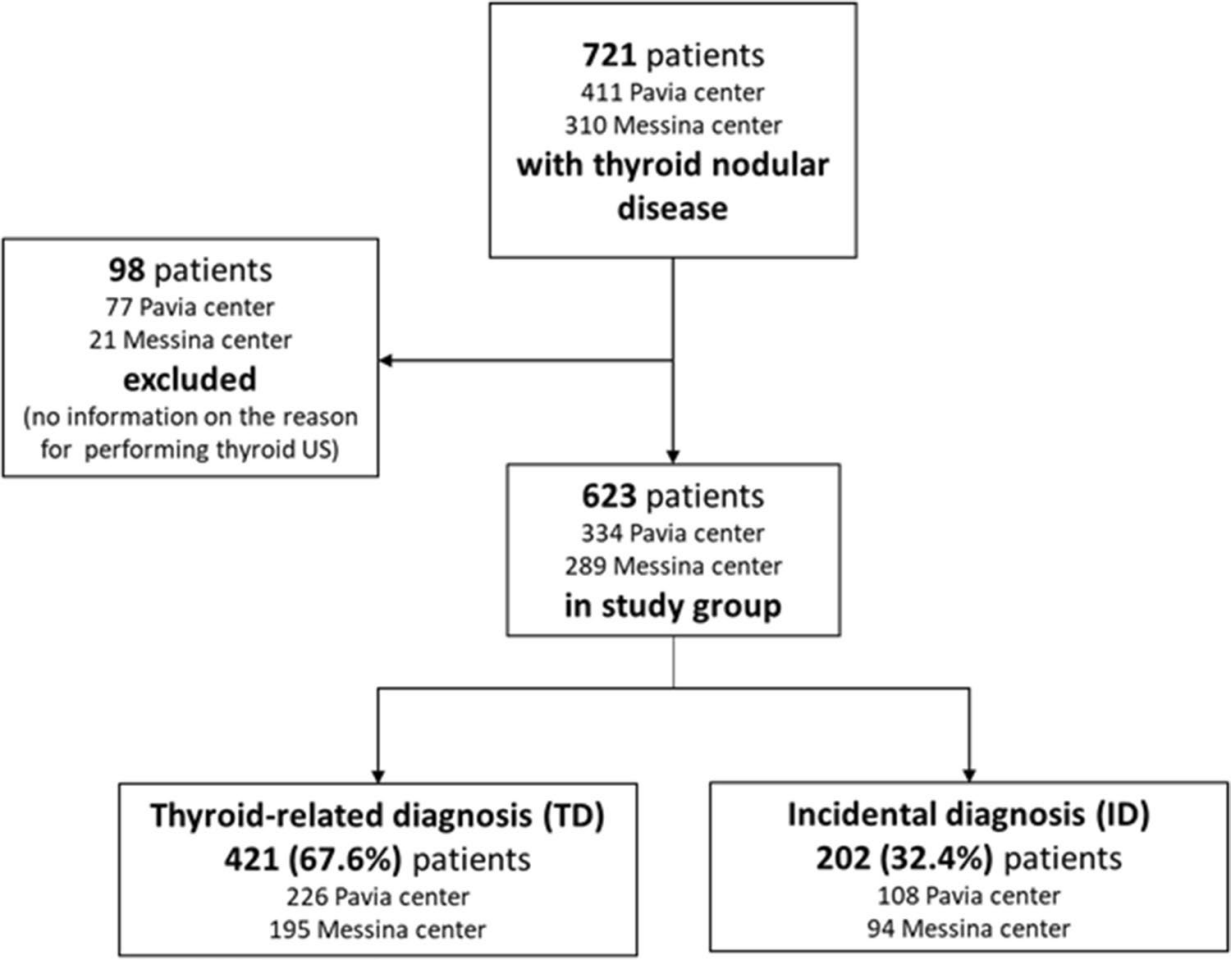
Flow diagram of the study. *US* ultrasound, *TD* thyroid-related disease, *ID* incidental diagnosis

As illustrated in the flow diagram in Fig. 1, the initial enrollment phase included 721 consecutive patients with TN (411 from Pavia center and 310 from Messina center). Due to the lack of information regarding the reason for performing a thyroid ultrasound, a total of 98 patients (77 from Pavia center and 21 from Messina center), were excluded from the final study group. The final study group encompassed 623 patients (334 from Pavia and 289 from Messina centers).

Considering the 623 included subjects, the mean age was 53.3 ± 15.4 years, mean BMI was 27.8 ± 11.5 kg/m^2^, and the majority of patients (452, 72.6% of the total) were females. The majority of patients (349 patients, 56% of the total) had at least one anamnestically retrieved cardiovascular and/or metabolic comorbidity (i.e., at least one among hypertension, type 2 diabetes mellitus, obesity, dyslipidemia, or cardiovascular diseases).

The TD group included 421 patients (67.6%), and the ID one 202 patients (32.4%).The distribution of patients in TD and ID groups was similar in the two centers (*p* = 0.960).

**Fig. 2:**
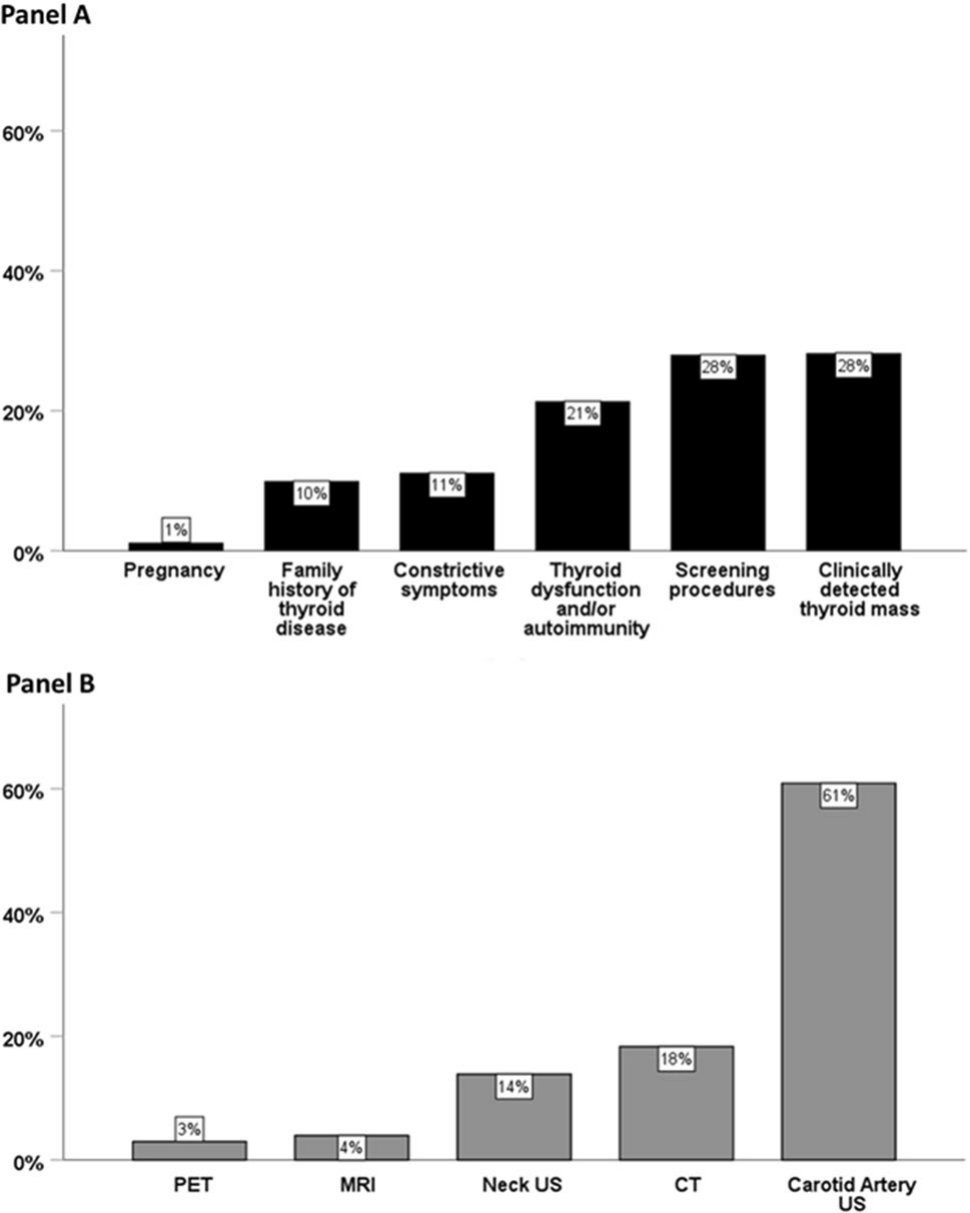
Histogram representing the distribution of the different causes for performing a thyroid US in TD group (Panel A) and ID group (Panel B). *TD* thyroid-related disease, *ID* incidental diagnosis, *US* ultrasound, *CT* computerized tomography, *MRI* magnetic resonance imaging, *PET* positron emission tomography

The prevalence of the different reasons leading to the performance of a thyroid ultrasound in TD and ID groups is represented in Fig. 2 Panels A and B, respectively. In detail, in the TD group, thyroid US was performed due to pregnancy in 5 patients (1%), a family history of thyroid disease in 42 patients (10%), constrictive symptoms in 47 patients (11%), thyroid dysfunction and/or autoimmunity in 90 patients (21%), screening procedures in 118 patients (28%), and the clinical detection of a thyroid mass in 119 patients (28%).

In the ID group, thyroid US was performed due to the incidental detection of a thyroid mass by Positron Emission Tomography (PET) scan in 6 cases (3.0%), MRI in 8 cases (4.0%), neck US not performed for a thyroid problem in 28 cases (14%), CT in 37 patients (18%), and carotid artery ultrasound (CA-US) in 123 patients (61%).

### Comparison of patients in the TD and ID groups

**Fig. 3:**
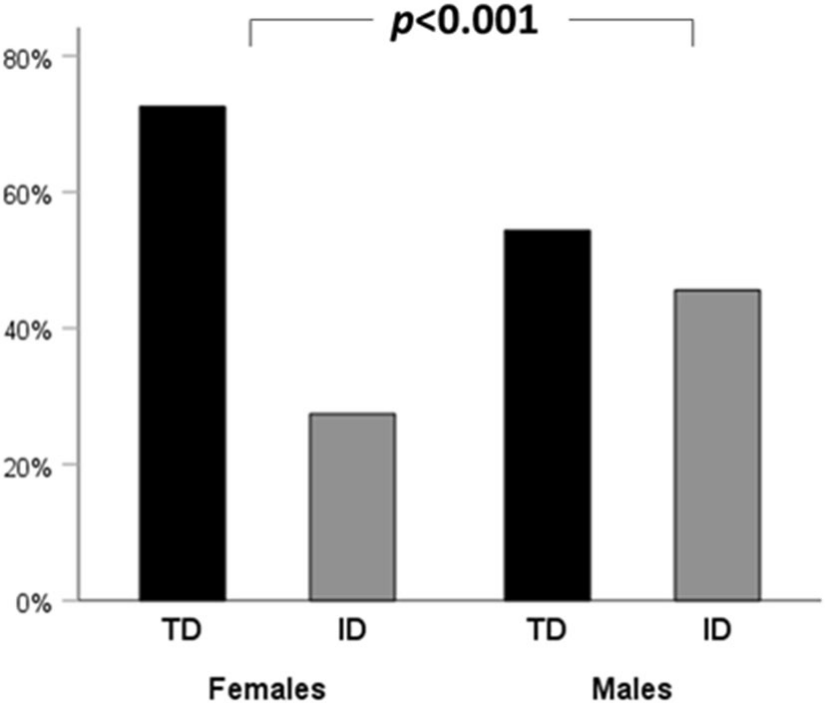
Histogram representing the percentage of patients in the TD and ID group after stratification for gender. *TD* thyroid-related disease; *ID* incidental diagnosis

As summarized in Table 1, patients in the ID group had a significantly higher male to female ratio, were significantly older, and had a significantly higher rate of cardiovascular comorbidities despite having a similar BMI, when compared to the TD group. Furthermore, thyroid volume and nodule maximum diameter were similar between the ID and TD groups, while a slightly higher number of nodules were observed in ID group when compared with TD group. Stratification according to gender showed that female patients had received a thyroid nodule diagnosis for thyroid-related reason in 72.6% of cases, while incidental diagnosis occurred only in 37.4% of cases. Among men, thyroid-related reasons lead to diagnosis only in 54.4% of cases, and incidental diagnosis was observed in 45.6% of cases (Fig. 3). Of note, in male patients older than 50 years, the incidental diagnosis accounted for more than half of cases (67 out of 127 patients 52.7%).

**Table 1:** Comparison between patients with an incidentally discovered thyroid nodule (ID) and patients diagnosed for thyroid-related reasons (TD)

	TD	ID	*p* value
*N*	421	202	
Age (years)	50.6 ± 15.5	58.9 ± 13.7	** < 0.001**
Sex M/F (% of males)	93/328 (22.1%)	78/124 (38.6%)	** < 0.001**
BMI (kg/m^2^)	27.8 ± 13.5	27.9 ± 5.2	0.893
Patients with cardiovascular/metabolic comorbidities (N, %)	200 (47.5%)	149 (73.8%)	** < 0.001**
Thyroid volume (ml)	20.5 ± 20.1	18.5 ± 13.9	0.319
Number of nodules (N (IQR))	3 (1–4)	3 (2–4)	**0.045**
Nodule maximum size (mm)	18.9 ± 13.6	19.8 ± 11.4	0.403

Bold indicates significant *p* values (lower than 0.05)

*IQR* inter-quartile range

As shown in Table 2, ID patients in whom a thyroid nodule was detected by CA-US had a higher prevalence of cardiovascular and metabolic comorbidities compared with the rest of the group. A non-significant trend toward an older age and a higher prevalence of male sex were observed.

**Table 2:** Comparison of patients with an incidental diagnosis of nodular goiter due to carotid artery ultrasound or due to other imaging

	id- carotid artery ultrasound	id- other imaging	*p* value
*N*	124	78	
Age (years)	60.3 ± 11.6	56.8 ± 16.3	0.069
Sex M/F (% of males)	54/69 (43.9%)	24/55 (30.4%)	0.054
BMI (kg/m^2^)	27.9 ± 4.5	27.9 ± 6.1	0.913
Patients with cardiovascular/metabolic comorbidities (*N*, %)	107 (87.0%)	42 (54.7%)	** < 0.001**

Bold indicates significant *p* values (lower than 0.05)

## Discussion

This study, performed in a large cohort of patients, had the aim of identifying the reasons leading to the diagnosis thyroid nodular disease. Since in the last years the use of neck imaging for several clinical conditions has become widespread, our hypothesis was that a relevant percentage of TN diagnosis is nowadays performed in non-endocrinological settings, the here-called “incidental diagnosis”. The results show that nearly one third of patients receive a first diagnosis of TN through diagnostic procedures which are unrelated to endocrinological conditions. In particular, elderly and male patients are more likely to receive an incidental diagnosis of TN (i.e., 43.6% of cases over 60 years old and 45.6% of male patients). The prevalence of cardiovascular and metabolic comorbidities was significantly higher in the ID group compared to the TD group, and such a difference was more relevant in male patients with increasing age.

This result should not be regarded as free of potential repercussions. Indeed, the first aspect requiring to be discussed is whether patients with nodular diseases should be considered as a homogenous category or whether more precise information could be retrieved if a stratification according to the cause leading to diagnosis would be performed.

Just to give an example, the here-reported results would confirm that patients with thyroid nodular disease are frequently characterized by the presence of cardiovascular and/or metabolic comorbidities, but on the other hand, they clearly demonstrate that patients receiving a thyroid-related or an incidental diagnosis display significant different rates for these comorbidities (47.5% versus 73.8%). This holds particularly true for elderly and male patients. It seems worth highlighting that, among the group with incidentally detected TN, carotid artery Doppler Ultrasound plays a major role, accounting for 61% of total incidental cases. Given that CA-US is more frequently performed in patients who are smokers, dyslipidemic, hypertensive, obese, with type 2 diabetes, and cardiovascular disorders, it seems clear the way in which diagnosis of TN is rendered has a huge impact on the clinical phenotype of these patients.

Several studies have identified cardiovascular and metabolic disorders and overweight/obesity as risk factors for the development of TN and cancer, sometimes with conflicting results, as many other studies have suggested a bidirectional association between TN and cardio-metabolic disorders [7, 13–20]. In particular a clinical association between insulin resistance, diabetes and thyroid nodular disease has been postulated [4–8]. In a large cohort of patients from a mild iodine deficient area, Buscemi and coworkers reported an increased prevalence of obesity/overweight in patients diagnosed with multiple TN and a significant correlation between TN and BMI, suggesting that an association exists between obesity, diabetes, and TN. However, no clear association emerged between TN and glycated hemoglobin, serum insulin levels, and homeostasis model assessment of insulin resistance (HOMA-IR) [6]. Another study by Aydoğan Y and coworkers failed to find any correlation between the presence and the number of TN and HOMA-IR and insulin levels, but suggested that patients with euthyroid TN might be at risk for cardiovascular disease based on the finding of increased arterial stiffness by pulse wave analysis [4].

The results of this study would suggest that different ratios of thyroid-related/incidental diagnosis of TN may strongly affect the strength of such an association. Thus, high rates of incidental diagnosis would lead to an enrichment of patients displaying a high burden of metabolic and cardiovascular comorbidities, suggesting that, according to the here-reported results, selection bias could be potentially introduced.

A second aspect stemming from the above results is that the health care professionals who perform the first diagnosis of thyroid nodular disease, nowadays, are often non-endocrinologists, such as cardiologists, radiologists, nuclear medicine physicians or vascular surgeons. Thus, a close collaboration between endocrinologists, other medical specialists and general practitioners, appears mandatory to allow a prompt and well-thought-out evaluation of patients with incidentally detected TN.

Our study has some limitations. Due to its retrospective nature, we could not actively look for asymptomatic or subclinical cardiovascular comorbidities in our patients.

Nevertheless, the high number of included patients coming from different geographical areas of Italy, together with the fact that both Centers involved in this study are second-level, hospital-based centers, should allow drawing firm conclusions regarding the potential impact of thyroid-related versus incidental diagnosis on the clinical and biochemical phenotype of patients with TN.

In conclusion, our results suggest that patients with TN represent an inhomogeneous population, demonstrating that stratification according to the diagnostic procedure leading to TN discovery, should be performed for a better epidemiological characterization of these patients, to avoid biases in the identification of risk factors and association of diseases.

## Data Availability

The datasets generated during the current study are available from the corresponding author on reasonable request.
